# The influence of underweight and obesity on the diagnosis and treatment of appendicitis in children

**DOI:** 10.1007/s00384-016-2614-6

**Published:** 2016-06-16

**Authors:** Marjolijn E. W. Timmerman, Henk Groen, Erik Heineman, Paul M. A. Broens

**Affiliations:** Department of Surgery, Division of Pediatric Surgery, University of Groningen, University Medical Center Groningen, Groningen, the Netherlands; Department of Epidemiology, University of Groningen, University Medical Center Groningen, Groningen, the Netherlands; Department of Surgery, Anorectal Physiology Laboratory, University of Groningen, University Medical Center Groningen, Groningen, the Netherlands

**Keywords:** Appendectomy, Appendicitis, Body mass index, Obesity, Underweight

## Abstract

**Purpose:**

The impact of lower body mass index (BMI) on appendicitis has never been addressed. We investigated whether different BMIs affect the diagnosis and treatment of appendicitis in children.

**Methods:**

The correlation between BMI and diagnosis accuracy and treatment quality was evaluated by retrospective analysis of 457 children diagnosed with appendicitis. Based on BMI percentiles, patients were classified as either underweight (*n* = 36), normal weight (*n* = 346), overweight (*n* = 59), or obese (*n* = 16). Diagnosis accuracy was measured by negative appendectomy rate, perforation rate, and number of consultations. Treatment quality was measured by complication rate and length of hospital stay.

**Results:**

Underweight patients had the highest negative appendectomy (OR 3.00, *P* = 0.008) and complication (OR 2.75, *P* = 0.041) rate. BMI did not influence perforation rate or number of consultations. Both underweight and obese patients stayed in the hospital longer than normal weight patients (regression coefficient 2.34, *P* = 0.001, and regression coefficient 9.40, *P* < 0.001, respectively).

Furthermore, in obese patients, the hospital stay after open appendectomy was prolonged compared to laparoscopic appendectomy (*P* < 0.001). No such differences were observed in patients with lower BMI.

**Conclusions:**

Underweight children are misdiagnosed more often, stay in hospital longer, and experience more postoperative complications than children of normal weight. Obesity is associated with longer hospital stays. Laparoscopic appendectomy might shorten the length of hospital stays in these patients. We conclude that in addition to obesity, underweight should also be considered a risk factor for children with appendicitis.

## Introduction

Appendectomy is one of the most common surgical interventions and is performed in approximately 1–4 in 1000 children per year [1]. Obesity was reported to negatively affect the time required for and the accuracy of the diagnosis of appendicitis in children [1–3]. Furthermore, obesity was found to affect wound healing due to delay in the recovery of mechanical strength, decreased wound collagen deposition, and an increased risk of inflammation [4]. Therefore, in adult patients, obesity also appears to impact the complication rate and length of hospital stay after appendectomy [5–7]. Nevertheless, reports to the contrary have also been published [2, 8].

Whereas obesity is a recognized risk factor for accurate diagnosis and surgical outcomes, the impact of a low BMI has not been addressed. In fact, underweight patients are often excluded from studies evaluating the influence of BMI on outcomes despite the fact that any abnormality, including underweight, may influence a patient’s health [9]. It was reported that underweight changes physiological processes and may, for instance, lead to malnutrition and osteoporosis [10], impaired functioning of the immune system [11], or disturbed wound healing [12]. Preoperative malnutrition was identified as an important predictor of poor clinical outcomes in adult patients undergoing gastrointestinal operations [13]. Surprisingly, little is known about the correlation between underweight and the diagnosis or surgical outcomes after appendicitis.

Surgical intervention in case of appendicitis involves different procedures, of which the open appendectomy and laparoscopic appendectomy are most frequently performed. The influence of BMI on the outcome of these two types of appendectomy is still under debate. Several studies demonstrated that a laparoscopic appendectomy associates with better outcomes in both obese children and adults in the sense of fewer complications and shorter hospital stays [6, 14, 15], while other studies reported no additional benefit from laparoscopy for obese patients compared to non-obese patients [16, 17].

Our first aim was to determine whether BMI, from low (underweight) to high (obesity), influenced the diagnosis of appendicitis in children. Our second aim was to study whether BMI influenced the outcome of the treatment of appendicitis. Our third aim was to analyze which operative technique had a favorable outcome in the different BMI categories.

## Materials and methods

### Patients

We compiled a database of 697 patients (5–18 years old), diagnosed with appendicitis, and who underwent an appendectomy. These patients were referred to the emergency department of University Medical Center Groningen, in the Netherlands, between January 2000 and September 2015.

We excluded 208 patients from the database of 697 patients due to lack of information on weight and/or height (the two parameters required to calculate BMI). Subsequently, we also excluded 32 patients who had a median laparotomy, since this technique is used if complications are expected to occur. Finally, 457 patients were selected for analyses.

The local medical ethics committee approved this study.

### Variables

To study the influence of BMI on the diagnosis of appendicitis, we analyzed the negative appendectomy rate, perforation rate, and number of consultations. The negative appendectomy rate was a measure for the number of patients misdiagnosed with appendicitis according to perioperative findings and/or pathological examination of the removed appendix. The perforation rate was based on the percentage of perforations visible during appendectomy. Consultations were performed by a surgeon in training under supervision of a pediatric or general surgeon. An additional consultation the next day was used for clinical reassessment during which laboratory and/or imaging investigations were repeated. For 55 patients, data were missing regarding the number of consultations required for diagnosing the patient.

To establish a possible correlation between BMI and its influence on the treatment of appendicitis, we investigated the complication rate and length of hospital stay. Data on the length of hospital stay were missing for eight patients. The postoperative complications taken into account were wound infections, abscess, fever lasting for more than 2 days after appendectomy, peritonitis developed after appendectomy, hematoma, bowel obstruction, urinary tract problems, postoperative ileus, readmission, and one or more reoperations.

BMI in children was corrected for age and gender and expressed in percentiles ranging from 0 to 100, where 0 stood for severe underweight and 100 for extreme obesity [18]. We investigated the following BMI groups: BMI percentile <5 (underweight, *n* = 36), BMI percentile 5–84 (normal weight, *n* = 346), BMI percentile 85–94 (overweight, *n* = 59), and BMI ≥ 95 percentile (obese, *n* = 16).

To establish possible correlations between BMI and the performed operative techniques, we compared the outcomes after laparoscopic and open appendectomy. Open appendectomy was performed mainly during the first years of this study, while a laparoscopic approach was used more often during the last few years. We, therefore, corrected our analyses for the year in which the appendectomy had been performed. Prophylactic antimicrobial treatment was the same for both operative techniques. Appendectomy was performed within 12 h of diagnosis, and the operation was not postponed until the next day. Data on the type of appendectomy used were available for 411 patients. All patients underwent standard clinical and laboratory assessment. Information on the use of ultrasound and CT scan was available for 402 patients.

To correct for variables in the multivariable analyses, we used information on the year the appendectomy had been performed and on age, gender, height, weight, operative technique, perforation status, C-reactive protein (CRP) levels, and leukocyte levels of the patients. The variables leukocytosis and increased CRP level were both corrected for age and gender.

### Statistical analysis

We used SPSS 22.0 for Windows (IBM SPSS Inc., Armonk, NY) for the statistical analyses of the data. A descriptive analysis was performed for all the variables. To test if variables were normally distributed, we used the Kolmogorov-Smirnoff and Shapiro-Wilk tests. For normally distributed continuous data, we used the independent-sample Student’s *t* test, and to analyze abnormally distributed continuous data, we used the Mann-Whitney *U* test. We used the chi-square test to compare categorical data. After the univariate analyses, we performed a multivariable analysis to correct for variables such as age and gender to create a better simulation of the actual clinical setting. The multivariable analyses used to determine diagnostic accuracy were corrected for year of appendectomy, age, and gender. Multivariable analyses used to determine treatment quality were corrected for year of appendectomy, age, gender, type of operative technique, perforation status, CRP level, and leukocyte level. We used binary logistic regression to estimate the odds ratio (OR) and 95 % confidence interval (95 % CI) for the binominal outcomes (negative appendectomy rate, perforation rate, number of consultations, and complication rate). Linear regression was used to estimate the regression coefficient (*B*) and 95 % CI for the continuous outcome (length of hospital stay). The BMI category “normal weight” was used as a reference value to which all the other BMI categories were compared. We considered *P* values below 0.05 statistically significant.

To investigate which operative technique had the most favorable outcome in terms of complications and length of hospital stay for the different BMI categories, we used an interaction term. The interaction term indicates whether the influence of one variable depends on the value of another variable, e.g., whether the influence of BMI on the occurrence of complications is different for BMI groups according to surgical technique. If the interaction term is significant (*P* < 0.05), it implies that BMI and the operative technique interact with each other and that therefore, these factors together have a different influence on the outcome of the analyses than separately.

## Results

### Characteristics of patients, diagnostic methods, and operative techniques

The patient characteristics per BMI group are presented in Table 1. Underweight children were significantly younger than children with normal weight (*P* = 0.001). There was no difference in gender distribution per BMI group.

**Table 1:** Patient characteristics, diagnostic methods, and operative techniques per BMI category

BMI category	Patient characteristics	
	Mean age (SD)	Gender
Underweight *n* = 36	11.0 (4.07)*	Girls 11 (31 %) Boys 25 (69 %)
Normal weight *n* = 346	13.2 (3.51)	Girls 162 (47 %) Boys 184 (53 %)
Overweight *n* = 59	13.0 (3.77)	Girls 29 (49 %) Boys 30 (51 %)
Obese *n* = 16	12.4 (3.61)	Girls 8 (50 %) Boys 8 (50 %)
BMI category	Diagnostic methods and operative techniques	
	Ultrasound	Operative techniques
Underweight *n* = 36	Performed 19 (63 %)**	LA 17 (53 %) OA 15 (47 %)***
Normal weight *n* = 346	Performed 121 (40 %)	LA 226 (73 %) OA 84 (27 %)
Overweight *n* = 59	Performed 20 (37 %)	LA 39 (72 %) OA 15 (28 %)
Obese *n* = 16	Performed 6 (50 %)	LA 10 (67 %) OA 5 (33 %)

The descriptive analyses of patient characteristics, diagnostic methods, and operative techniques were all corrected for the year in which the patient had been diagnosed with appendicitis

*LA* laparoscopic appendectomy, *OA* open appendectomy

*P = 0.001 in underweight patients in comparison to normal weight patients

**P = 0.023 in underweight patients in comparison to normal weight patients

***P = 0.005 in underweight patients in comparison to normal weight patients

The clinical and laboratory assessment was the same for each BMI group. Nevertheless, an ultrasound was performed almost twice as often in the underweight group than in the other BMI groups (*P* = 0.023) (Table 1). Age and gender had no significant influence on the use of ultrasound.

In most BMI groups, laparoscopic appendectomy had been performed more often than open appendectomy, except in the case of the underweight group (*P* = 0.005). In these patients, laparoscopic appendectomy was performed equally often as open appendectomy.

### Influence of BMI on diagnosing appendicitis

The descriptive analysis showed that underweight children had a negative appendectomy more often than patients with normal weight (28 versus 18 %, respectively). Multivariable analysis confirmed that underweight children had three times higher risk of a negative appendectomy than normal weight patients (*P* = 0.008) (Table 2). Furthermore, the multivariable analysis with extra correction for ultrasound use showed that both underweight and the use of ultrasound influenced the negative appendectomy rate (*P* = 0.008 and *P* = 0.004, respectively). Underweight, however, had a negative influence, whereas ultrasound influenced the negative appendectomy rate positively. We found that obese children had a negative appendectomy rate of 25 %, although this was not significant when multivariable analysis was performed (*P* = 0.72). Apart from BMI, older children had a higher chance of a negative appendectomy (OR 1.11, 95 % CI 1.03–1.20, *P* = 0.005). Similarly, girls had a higher chance of experiencing a negative appendectomy than boys (OR 2.38, 95%CI 1.43–3.98, *P* < 0.001).

**Table 2:** The influence of BMI on diagnosing appendicitis

BMI category	Negative appendectomies			
	Number of patients	OR	95 % CI	*P*
Underweight	10/36 (28 %)*	3.00	1.29–6.94	0.008*
Normal weight	61/346 (18 %)	Ref.	Ref.	–
Overweight	6/59 (10 %)	0.504	0.203–1.25	0.13
Obese	4/16 (25 %)	1.76	0.523–5.90	0.72
BMI category	Perforations			
	Number of patients	OR	95 % CI	*P*
Underweight	11/36 (31 %)	1.62	0.748–3.52	0.24
Normal weight	70/346 (20 %)	Ref.	Ref.	–
Overweight	7/59 (12 %)	0.523	0.227–1.20	0.14
Obese	3/16 (18 %)	0.872	0.241–3.160	0.87
BMI category	Consultations			
	Number of patients	OR	95 % CI	*P*
Underweight	1 consultation 30/30 (100 %) >1 consultations 0/30 (0 %)	< 0.001	0.00–0.00	0.99
Normal weight	1 consultation 267/306 (87 %) >1 consultations 39/306 (13 %)	Ref.	Ref.	–
Overweight	1 consultation 48/54 (89 %) >1 consultations 6/54 (11 %)	0.855	0.343–2.13	0.92
Obese	1 consultation 10/12 (83 %) >1 consultations 2/12 (17 %)	1.41	0.295–6.68	0.63

The influence of BMI on diagnosing appendicitis measured in terms of the number of negative appendectomies, perforations, and consultations required to diagnose the patient. The multivariate regression analysis was corrected for year, age, and gender

*Ref*. the reference group, i.e., the group of patients with normal weights with which each one of the other three BMI categories was compared, *OR* odds ratio, *95 % CI* 95 % confidence interval

*P < 0.05

Moreover, a perforated appendicitis was observed more often in underweight children (31 %) than in normal weight patients (20 %), although this difference was not significant (*P* = 0.24) (Table 2). Overweight and obesity also did not have a significant influence on perforation rate.

There was no correlation between BMI and the number of consultations required for diagnosing appendicitis (Table 2).

### Influence of BMI on treatment of appendicitis

Underweight and obese patients had the highest complication rate after appendectomy, i.e., 25 % in both cases (Table 3). The multivariable analyses, however, showed that the difference in complication rate was only significant for the underweight patients, since they had three times higher risk of complications compared to normal weight children (*P* = 0.041). Underweight patients experienced the following complications: wound infection (6 %), abscess (8 %), peritonitis (3 %), fever (5 %), bowel obstruction (3 %), urinary tract problem (6 %), postoperative ileus (3 %), readmission (6 %), and reoperation (6 %).

**Table 3:** The influence of BMI on the treatment of appendicitis

BMI category	Complications			
	Number of patients	OR	95 % CI	*P*
Underweight	9/36 (25 %)	2.75	1.03–7.35	0.041*
Normal weight	52/346 (15 %)	Ref.	Ref.	–
Overweight	5/59 (9 %)	0.655	0.217–1.98	0.44
Obese	4/16 (25 %)	2.08	0.391–11.0	0.39
BMI category	Length of hospital stay			
	Median of days (min-max)	*B*	95 % CI	*P*
Underweight	4.5 days (1–24)	2.34	0.797–3.89	0.001*
Normal weight	3.0 days (0–23)	Ref.	Ref.	–
Overweight	2.0 days (1–13)	−0.064	−1.21–1.08	0.80
Obese	3.5 days (0–55)	9.10	6.55–11.7	<0.001**

The influence of BMI on the treatment of appendicitis as measured in terms of complication rate and length of hospital stay. The multivariate regression analysis was corrected for year, age, gender, operative technique, perforation status, CRP levels, and leukocyte levels

*Ref*. the reference group, i.e., the group of patients with normal weights with which each one of the other three BMI categories was compared, *OR* odds ratio, *95 % CI* 95 % confidence interval, *B* regression coefficient

*P < 0.05; **P < 0.001

Univariate and multivariable analyses revealed that underweight and obese patients needed to stay in the hospital significantly longer than patients with normal weight: a median hospital stay of 4.5 and 3.5 days compared to 3.0 days (*P* = 0.001 and *P* < 0.001), respectively (Table 3).

### Influence of BMI on surgical outcome in correlation to operative techniques

First, we studied whether there was a difference in surgical outcome after laparoscopic and open appendectomy in general, prior to subdividing the children into different BMI categories. We found that 17 % of the children experienced complications after laparoscopic appendectomy and 13 % after open appendectomy (*P* = 0.51). For both laparoscopic and open appendectomy, the median length of hospital stay was 3.0 days (*P* = 0.81).

Subsequently, we analyzed the influence of the operative technique among different BMI groups to determine the most suitable technique for each group. We observed that the median length of hospital stay and complication rate of underweight patients who had undergone either laparoscopic or open appendectomy was not significantly different (*P* = 0.79 and *P* = 0.059, respectively). Similarly, the length of hospital stay and complication rate were not significantly different for overweight patients (*P* = 0.58 and *P* = 0.65, respectively).

**Fig. 1:**
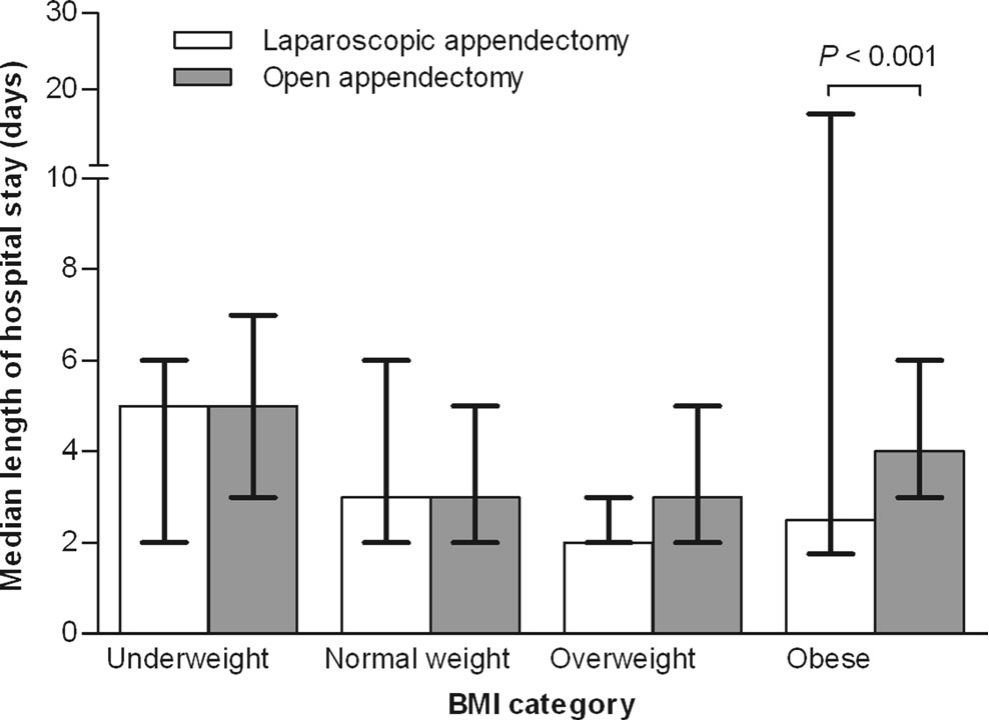
Influence of BMI on the length of hospital stay after laparoscopic and open appendectomy. Length of hospital stay was measured in terms of median days with interquartile range

We did, however, find a significant difference between the lengths of hospital stays in obese patients who had undergone either laparoscopic or open appendectomy (*P* < 0.001). After laparoscopic appendectomy, obese patients stayed in the hospital for a median of 2.5 days (interquartile range 1.75–16.75), compared to 3.0 days in patients with normal weights. Obese patients, who had undergone open appendectomy, stayed in the hospital for a median of 4.0 days (interquartile range 3.0–6.0), compared to patients with normal weights who stayed in the hospital for 3.0 days (Fig. 1). There was no difference in complication rate between obese and normal weight children when operative technique was taken into account (*P* = 0.99).

## Discussion

We demonstrated that underweight significantly hampers the accuracy of diagnosing appendicitis by increasing the negative appendectomy rate. Furthermore, both underweight and obesity seemed to negatively influence the treatment of appendicitis by increasing the complication rate and length of hospital stay. Finally, our data confirmed that laparoscopic appendectomy should be the treatment of choice for obese children, since it is associated with a shorter stay in hospital.

Multiple factors are known to influence negative appendectomy rates, for instance age and gender [19], as was confirmed by our study. Therefore, to properly investigate the correlation between BMI and negative appendectomy rate, we corrected our analysis for these factors. We found that underweight patients had a three times higher chance of a negative appendectomy than normal weight children. Other BMIs had no significant influence on the negative appendectomy rate. The negative appendectomy rate can also be influenced by the number and type of diagnostic tools used to diagnose appendicitis. In this study, patients from all four BMI groups were given a standard clinical and laboratory assessment. The underweight group, however, was examined more often by ultrasound than children from other BMI groups. Even when corrected for the possible influence of year of appendectomy, age, gender, and the use of ultrasound, we found that underweight children, independent of all those factors, still had a significantly higher risk of a negative appendectomy. It is important to note that underweight does not seem to influence the accuracy of ultrasound [20, 21]. We conclude, therefore, that even though underweight children were examined more extensively, they were nevertheless misdiagnosed with appendicitis more often than children from the other BMI groups. Although we cannot explain the aforementioned finding, we think it could be caused by the fact that children with underweight are more sensitive to abdominal examination. Consequently, the physician might think that underweight patients experience more pain during examination than normal weight and obese children. The alternative diagnoses for the children with negative appendectomies were not further investigated in this study.

Delay in an accurate diagnosis and the consequent delay of treatment results in perforation of the appendix. According to the literature, obesity may increase perforation rate [2]. We did not observe an increased perforation rate in any of the BMI groups. The discrepancy with another study [2] could possibly be explained by the differences in the statistical analyses used. We analyzed four different BMI groups, whereas Blanco et al., for instance, investigated two groups of patients: obese and non-obese [2]. Additionally, various hospitals use different methods to diagnose appendicitis, which may influence the time invested in accurate diagnosis and, therefore, perforation rate [22].

We found that the number of consultations required to diagnose appendicitis was not influenced by BMI. Our study is corroborated by a previous analysis where no significant difference between obese and non-obese patients was found in terms of consultations required to diagnose and perforation rate [17].

The potential influence of BMI on the complication rate after appendectomy is still a subject of debate. We found that in comparison to normal weight children, obese children experience a relatively large number of complications after appendectomy. This difference was, however, not significant, due probably to the small number of obese patients included in our study. We did find a significant difference in complication rate between underweight and normal weight children, i.e., underweight children had an almost three times higher risk of complications. Therefore, alongside obesity, one should also consider underweight as a risk factor for complications. Although interesting, we did not analyze in detail to which type of complications underweight children were prone, as the groups of patients with different complications were too small.

Quality of treatment can also be measured by considering the length of hospital stays. We found that hospital stays were significantly longer in both obese and underweight children, due possibly to their higher complication rates, although other factors might also have influenced the length of hospital stays. Staying for an additional 1–2 days in case of patients with underweight and obesity is associated with higher financial costs, which implies economic consequences.

Furthermore, we also found that obesity was associated with significantly prolonged length of hospital stays in patients who had undergone open appendectomy, rather than in case of laparoscopic appendectomy. We conclude, therefore, that laparoscopic appendectomy is more favorable for obese pediatric patients than open appendectomy, as has been reported for obese adults and children [6, 14, 15]. Mason et al. postulated that the advantage of the laparoscopic technique in obese patients is entirely due to wound and wound-related complications [23]. In line with this, since obese children often suffer from diabetes, the process of wound healing is worse than that in children with normal weight or underweight. It would be interesting to investigate in a follow-up study whether the occurrence of complications and length of hospital stays are increased in a subpopulation of obese children with diabetes.

## Final conclusion

In this study, we demonstrate that underweight increases the negative appendectomy rate significantly and that it should be considered a risk factor for the misdiagnosis of appendicitis. Since both underweight and obese patients showed a tendency toward having increased complication rates and longer hospital stays, we conclude that both underweight and obesity negatively influence the outcome of appendectomy. Since we found that age as well as gender influenced the chance of having a negative appendectomy, we conclude that BMI is not a single factor affecting the diagnosis and treatment of appendicitis. Lastly, laparoscopic appendectomy is recommended in case of obese children in order to decrease the length of hospital stays.
